# Neurotropic Threat Characterization of *Burkholderia pseudomallei* Strains

**DOI:** 10.3201/eid2101.131570

**Published:** 2015-01

**Authors:** Jodie Morris, Anne Fane, Catherine Rush, Brenda Govan, Mark Mayo, Bart J. Currie, Natkunam Ketheesan

**Affiliations:** James Cook University, Townsville, Queensland, Australia (J. Morris, A. Fane, C. Rush, B. Govan, N. Ketheesan); Menzies School of Health Research, Darwin, Northern Territory, Australia (M. Mayo, B.J. Currie);; Royal Darwin Hospital, Darwin (B.J. Currie)

**Keywords:** neurologic, melioidosis, Burkholderia pseudomallei, route of infection, virulence, neurotropism, bacteria, central nervous system, bioterrorism, melioidosis

## Abstract

The death rate for neurologic melioidosis is high. Whether certain *Burkholderia pseudomallei* strains are more likely than other strains to cause central nervous system infection and whether route of infection influences the neurotropic threat remain unclear. Therefore, we compared the virulence and dissemination of Australian clinical isolates collected during October 1989–October 2012 from patients with neurologic and nonneurologic melioidosis after intranasal and subcutaneous infection of mice in an experimental model. We did not observe neurotropism as a unique characteristic of isolates from patients with neurologic melioidosis. Rather, a distinct subset of *B. pseudomallei* strains appear to have heightened pathogenic potential for rapid dissemination to multiple tissues, including the central nervous system, irrespective of the infection route. This finding has valuable public health ramifications for initiating appropriate and timely therapy after exposure to systemically invasive B. pseudomallei strains. Increasing understanding of *B. pseudomallei* pathology and its influencing factors will further reduce illness and death from this disease.

## Abstract 2

Unique virulence factors of certain strains facilitate central nervous system invasion, regardless of infection route.

Melioidosis is caused by the gram-negative bacterium *Burkholderia pseudomallei*. It incorporates a wide spectrum of clinical disease that ranges from severe, rapidly fatal, invasive disease to asymptomatic latent infection; thus, diagnosis is immensely challenging (*1*). No vaccine against melioidosis is currently available. The ability of *B. pseudomallei* to cause severe, rapidly fatal, invasive infections and to persist in the environment for extended periods, plus its intrinsic resistance to many antibacterial drugs, make *B. pseudomallei* a desirable candidate for use as a bioterrorism agent (*1*). Furthermore, *B. pseudomallei* can invade host cells, including macrophages, neutrophils, and other cells of the immune system, and persist within them (*2*,*3*). Without appropriate drug therapy, the death rate for melioidosis can exceed 90% (*4*,*5*).

Neurologic abnormalities occur in 3%–5% of melioidosis cases, and more than one quarter of those are fatal (*5*–*8*). Many similarities have been described regarding the clinical features of neurologic melioidosis in naturally infected animals and humans and in animal models infected with *B. pseudomallei* (*6*,*9*–*15*). Cranial nerve palsies and unilateral limb weakness are frequently described in patients with neurologic melioidosis (*6*,*7*,*9*,*13*,*14*). Flaccid paraparesis, commonly documented in animals with *B. pseudomallei* infection, also has been reported in humans (*6*,*10*,*15*). Often, microscopic and macroscopic abscesses are evident, and a predilection of *B. pseudomallei* for the brainstem and spinal cord has been suggested (*9*,*12*–*14*).

In contrast to patients with other forms of melioidosis, those with melioidosis with central nervous system (CNS) involvement are less likely to have predisposing risk factors, such as diabetes and chronic lung disease (*6*,*8*,*9*). Relatively little is known about the potential for different *B. pseudomallei* strains to cause severe disease, including whether particular strains are more likely to cause neurologic sequelae or whether CNS involvement is a consequence of the mode of delivery of *B. pseudomallei*. Neurologic melioidosis can result from direct invasion or through hematogenous spread (*3*,*6*,*11*,*16*,*17*). Initial suggestions that neurologic melioidosis might result from damage from immune or toxin-mediated mechanisms (*6*,*12*) has been supplanted by the recognition that direct invasion of brain and spinal cord by bacteria is evident on histologic examination of samples from case-patients who died (*16*). Furthermore, direct invasion of the brain by *B. pseudomallei* was recently demonstrated in an experimental model of melioidosis meningitis after delivery of intracellular bacteria by CNS-infiltrating CD11b^+^ immune cells (*3*).

Given the high rate of death from neurologic melioidosis, interest is increasing in improving understanding of its pathogenesis, particularly the potential for different *B. pseudomallei* isolates to cause neurologic melioidosis and the influence of the route of transmission on entry into and dissemination within the CNS. Therefore, using a well-characterized animal model of melioidosis (*18*) and clinical isolates of *B. pseudomallei* collected in the Northern Territory, Australia, during October 1989–October 2012 (*7*), we sought to determine whether strains isolated from patients with neurologic melioidosis (neurologic isolates) showed higher virulence levels and neurotropism than isolates from patients with nonneurologic melioidosis (nonneurologic isolates) after respiratory and percutaneous exposure. 

## Methods

### *B. pseudomallei* Isolates

Eleven *B. pseudomallei* clinical isolates were analyzed. Six of these isolates were from patients with moderate to severe neurologic melioidosis, and 5 were from patients with nonneurologic melioidosis, both disseminated and localized cases (Table 1).

**Table 1:** Clinical features of *Burkholderia pseudomallei* strains isolated from patients with neurologic and nonneurologic melioidosis and their virulence in C57BL/6 and BALB/c mice, Northern Territory, Australia, October 1989–October 2012*

MSHR ID no.	Age, y/sex	Clinical feature	Risk factor	ID_50_ in mice (CFU)†				
				Intranasal			Subcutaneous	
				C57BL/6	BALB/c		C57BL/6	BALB/c
Neurologic								
668‡	53/M	Severe neurologic signs	None	4.1 × 10^4^	2.9 × 10^2^		7.1 × 10^3^	<10
305§	64/M	Severe neurologic signs	Alcohol use	2.6 × 10^2^	2.6 × 10^2^		3.7 × 10^4^	<10
62‡	24/M	Severe neurologic signs	None	2.2 × 10^2^	6.3 × 10^1^		2.4 × 10^2^	<10
435‡	37/M	Severe neurologic signs	Kava	5.0 × 10^2^	1.3 × 10^2^		<10	<10
1153§	60/M	Severe neurologic signs	Diabetes mellitus	1.7	<10		<10	<10
3709‡	14/M	Moderate neurologic signs	None	4.4 × 10^4^	1.8 × 10^4^		2.2 × 10^2^	1.3 × 10^2^
Nonneurologic								
1655‡	61/F	Chronic pulmonary	Bronchiectasis	>10^8^	>10^8^		>2 × 10^8^	>2 × 10^8^
465§	67/M	Septicemia	Diabetes mellitus, chronic obstructive pulmonary disease	1.3 × 10^5^	1.1 × 10^3^		8.3 × 10^5^	<10
2138‡	49/F	Septicemia	Diabetes mellitus	3.6 × 10^3^	<10		4.2 × 10^3^	1.2 × 10^1^
346‡	49/M	Chronic pulmonary	Alcohol use	8.5 × 10^4^	6.0 × 10^4^		6.0 × 10^5^	7.0 × 10^5^
543‡	22/F	Skin ulcer	None	2.9 × 10^2^	8.5 × 10^1^		1.2 × 10^1^	<10

*ID_50_, 50% infectious dose; MSHR ID, neurologic isolate identification. †ID_50_ determined after intranasal and subcutaneous infection of C57BL/6 and BALB/c mice ‡Nonfatal. §Fatal.

### Animals

The C57BL/6–BALB/c mouse model is a well-characterized model of differential susceptibility to *B. pseudomallei* (*18*). We included C57BL/6 and BALB/c mice to enable comparison of disease progression within immunocompetent and immune-impaired hosts, respectively. We purchased 8- to 12-week-old BALB/c and C57BL/6 mice from the Small Animal Breeding Facility, James Cook University (Townsville, QLD, Australia). Experiments were conducted in accordance with National Health and Medical Research Council guidelines and were approved by the institutional ethics committee (A1500).

### Preparation and Delivery of *B. pseudomallei* Isolates

*B. pseudomallei* isolates were cultured in tryptic soy broth at 37°C to logarithmic phase. After washing in phosphate-buffered saline (PBS), pH 7.2, bacteria were suspended to 10^8^ CFU/mL. Serial 10-fold dilutions were prepared in sterile PBS to obtain required infectious doses. Doses were confirmed retrospectively by plating serial dilutions onto Ashdown agar (AA). To mimic natural routes of infection, intranasal or subcutaneous routes were used for inoculation with *B. pseudomallei* by previously described methods (*18*).

### Determination of 50% Infectious Dose

Groups of 5 mice were inoculated intranasally and subcutaneously at 10-fold increasing doses of *B. pseudomallei*, ranging from 10^0^ CFU to 10^7^ CFU (*18*). Survival was monitored for 21 days, and moribund mice were euthanized. At necropsy, organs were observed for presence of visible abscesses. Spleens were homogenized and plated on AA to confirm the presence of *B. pseudomallei* in mice that died from their infection within the experiment period. Mice that survived to 21 days after infection were euthanized and underwent necropsy to determine whether abscesses were visible in spleen and liver. For mice infected subcutaneously, the subcutaneous adipose tissue at the site of infection was also assessed. Tissue homogenates were cultured on AA to confirm the presence or absence of persistent *B. pseudomallei* infection. We determined the 50% infectious dose (ID_50_) from the total number of mice that either died of their infection or had evidence of persistent infection 21 days postinfection (dpi) using a modified version of the Reed and Meunch method (*19*). ID_50_ for neurologic and nonneurologic isolates are expressed as mean log_10_ CFU ± the standard error of the mean (SEM). Virulence, as defined by the ID_50_ of *B. pseudomallei* isolates derived from patients with neurologic and nonneurologic melioidosis, were compared in BALB/c and C57BL/6 mice after both intranasal and subcutaneous infection.

### Determination of Bacterial Load

At specified time points, 5 mice were euthanized by cardiac puncture, and blood was collected into sterile tubes containing lithium heparin. Bacterial load in blood was determined by plating serial dilutions of whole blood in PBS onto AA and counting colonies after 24–48 h incubation at 37°C. Immediately after collection of blood, the liver, spleen, lung, lymph nodes (cervical and inguinal), brain, and nasal-associated lymphoid tissue were aseptically excised. Tissue bacterial load was determined by homogenizing tissue in 1 mL of PBS and plating serial dilutions onto AA for colony counts. The detection limit of bacteria in tissues was 2 CFU. Data were expressed as the mean log_10_ CFU ± SEM.

### Statistical Analysis

For statistical analysis, we used Graphpad Prism version 6 (http://www.graphpad.com). Kaplan–Meier survival curves were used to compare susceptibility to infection with *B. pseudomallei* isolates after infection by different routes. ID_50_ for neurologic and nonneurologic *B. pseudomallei* isolates were compared by using Student *t* test. Bacterial loads in organs after *B. pseudomallei* infection by different routes were tested for significance using 1-way analysis of variance based on normally distributed sets of data. Comparisons were considered to be significant at p<0.05.

## Results

Although neurologic isolates tended to be more virulent (lower ID_50_) than nonneurologic isolates after intranasal infection, this finding did not reach statistical significance (Table 1). Consistent with previous evidence for differential susceptibility, C57BL/6 mice demonstrated greater resistance than BALB/c mice to *B. pseudomallei* infection, as indicated by their 10-fold higher ID_50_ (*18*).

Signs of CNS involvement (i.e., head tilt and/or circling, difficulty walking, limb paresis) developed after intranasal infection of BALB/c and C57BL/6 mice, typically 8–12 dpi. This feature was not unique to neurologic isolates; head tilt and limb paralysis also were observed in mice infected with non-neurologic isolates. The development of neurologic signs corresponded with bacterial loads in brain, reaching >10^3^ CFU. However, the data suggest that development of neurologic signs did not depend on the initial infectious dose because serial increases in the inoculating dose failed to cause a stepwise increase in number of C57BL/6 mice with neurologic signs (Table 2). Similar trends were observed after intranasal infection of BALB/c mice (data not shown).

**Table 2:** Development of signs of neurologic involvement* in C57BL/6 mice after intranasal infection with *Burkholderia pseudomallei* strains isolated from patients with neurologic and non-neurologic melioidosis, Northern Territory, Australia, October 1989–October 2012

**MSHR ID no.†**	**Inoculating dose, CFU**	**No. mice with neurologic signs/total mice infected**
**Neurologic**		
** 668**	2.9 × 10^3^	1/5
	2.9 × 10^4^	0/5
	2.9 × 10^5^	2/5
	2.9 × 10^6^	2/5
** 305**	2.6 × 10^3^	0/5
	2.6 × 10^4^	1/5
	2.6 × 10^5^	3/5
	2.6 × 10^6^	0/5
** 62**	2.2 × 10^3^	1/5
	2.2 × 10^4^	1/5
	2.2 × 10^5^	2/5
	2.2 × 10^6^	1/5
** 435**	3.0 × 10^3^	0/5
	3.0 × 10^4^	3/5
	3.0 × 10^5^	2/5
	3.0 × 10^6^	2/5
** 1153**	5.3 × 10^1^	1/5
	5.3 × 10^2^	3/5
	5.3 × 10^3^	0/5
	5.3 × 10^4^	1/5
** 3709**	2.2 × 10^3^	0/5
	2.2 × 10^4^	0/5
	2.2 × 10^5^	0/5
	2.2 × 10^6^	0/5
**Non-neurologic**		
** 1655**	1.1 × 10^4^	0/5
	1.1 × 10^5^	0/5
	1.1 × 10^6^	0/5
	1.1 × 10^7^	0/5
** 465**	6.6 × 10^3^	0/5
	6.6 × 10^4^	0/5
	6.6 × 10^5^	2/6
	6.6 × 10^6^	1/6
** 2138**	2.4 × 10^2^	0/5
	2.4 × 10^3^	0/5
	2.4 × 10^4^	1/5
	2.4 × 10^5^	2/4
** 346**	4.2 × 10^3^	0/5
	4.2 × 10^4^	0/5
	4.2 × 10^5^	2/5
	4.2 × 10^6^	0/5
** 543**	9.4 × 10^2^	1/5
	9.4 × 10^3^	1/5
	9.4 × 10^4^	3/5
	9.4 × 10^5^	2/5

*Head tilt, difficulty walking, limb paresis. **†MSHR ID, neurologic isolate identification.**

After subcutaneous infection with *B. pseudomallei* isolates, ID_50_ ranged from <10 CFU to >2 × 10^8^ CFU in BALB/c and C57BL/6 mice. Consistent with intranasal infection, BALB/c mice were more susceptible than C57BL/6 mice to subcutaneous infection with *B. pseudomallei*, as indicated by their 10–100-fold lower ID_50_. Similar to findings after intranasal infection, neurologic isolates tended to be more virulent (lower ID_50_) than nonneurologic isolates after subcutaneous infection, although this finding did not reach statistical significance.

Mean ID_50_ was comparable for C57BL/6 mice after intranasal (3.2 × 10^4^ CFU) or subcutaneous (9.3 × 10^4^ CFU) infection with neurologic isolates. Similarly, mean ID_50_ for BALB/c mice did not differ between intranasal (7.6 × 10^2^ CFU) and subcutaneous (8.1 × 10^2^ CFU) infection. Neurologic isolates appeared to be more infectious for BALB/c mice when delivered subcutaneously rather than intranasally (p = 0.04). However, we did not observe this phenomenon for C57BL/6 mice.

**Figure 1:**
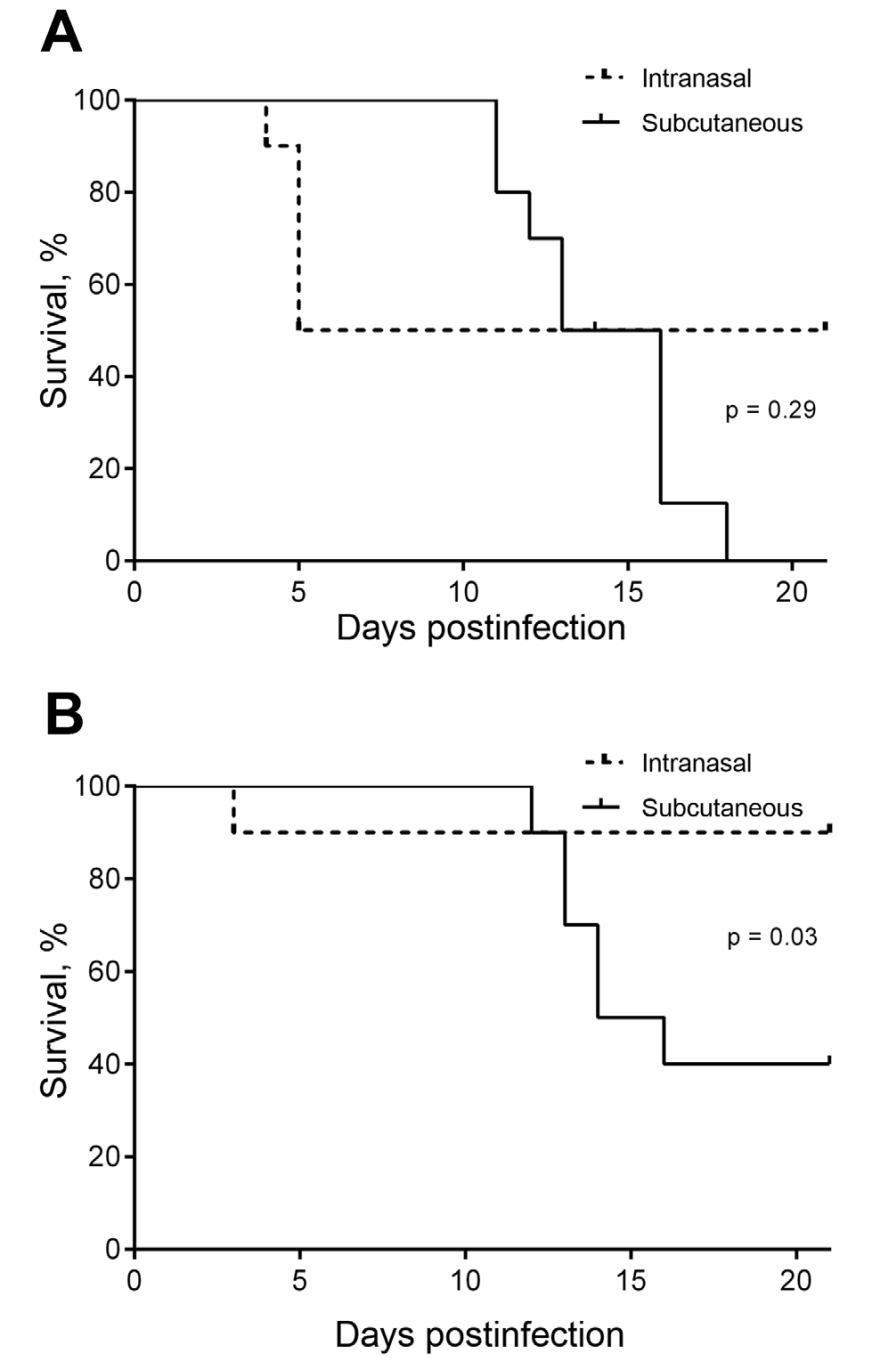
Comparison of survival after intranasal and subcutaneous infection of BALB/c mice with equivalent doses of the neurologic *Burkholderia pseudomallei* isolates MSHR435 (5 × 10^2^ CFU) (A) and MSHR1153 (4.5 × 10^2^ CFU) (B), Northern Territory, Australia, October 1989–October 2012. This inoculation dose was >50× the 50% infectious dose for MSHR435 and MSHR1153, delivered by intranasal or subcutaneous inoculation. Data are expressed as percentage survival; 10 mice were monitored within each group for 21 days postinfection.

We conducted a second series of studies to compare early bacterial load kinetics within tissues after intranasal and subcutaneous infection with *B. pseudomallei*. The purpose of these studies was to determine whether infection of the brain occurred more rapidly after intranasal than subcutaneous infection with isolates from neurologic melioidosis. The neurologic isolates, MSHR435 and MSHR1153, were associated with severe neurologic melioidosis whereby the CNS was suspected to be the primary site of infection and therefore represent isolates with high potential for direct CNS invasion after intranasal exposure in an animal model. BALB/c mice were inoculated with equivalent doses of *B. pseudomallei* intranasally or subcutaneously. Survival was monitored for 21 days. In addition, bacterial loads were determined in nasal-associated lymphoid tissue, draining lymph nodes (cervical or inguinal), blood, lung, brain, spleen, liver, and subcutaneous adipose tissue at the site of infection at 1, 3, and 7 dpi. BALB/c mice tended to be more susceptible to MSHR435 when infected subcutaneously; the rate of death reached 100% within 18 days, although this finding was not statistically significant (p = 0.29) (Figure 1, panel A**)**. However, the death rate for infection of BALB/c with MSHR1153 was significantly higher after subcutaneous inoculation; hind leg paresis developed within the second week after infection that necessitated euthanasia of 6 of the 10 mice (p = 0.03, Figure 1, panel B). In contrast to subcutaneous infection, greater variability in disease progression was associated with intranasal inoculation of mice with *B. pseudomallei*, ranging from rapid systemic dissemination in some mice to low-level persistence in the respiratory tract with potential for clearance within a week after exposure. Infection was established in 100% of mice when MSRH435 or MSHR1153 was delivered subcutaneously. Consequently, the differences in overall death rates for mice after intranasal and subcutaneous infection reflect the variability in dissemination of *B. pseudomallei* after respiratory exposure.

**Figure 2:**
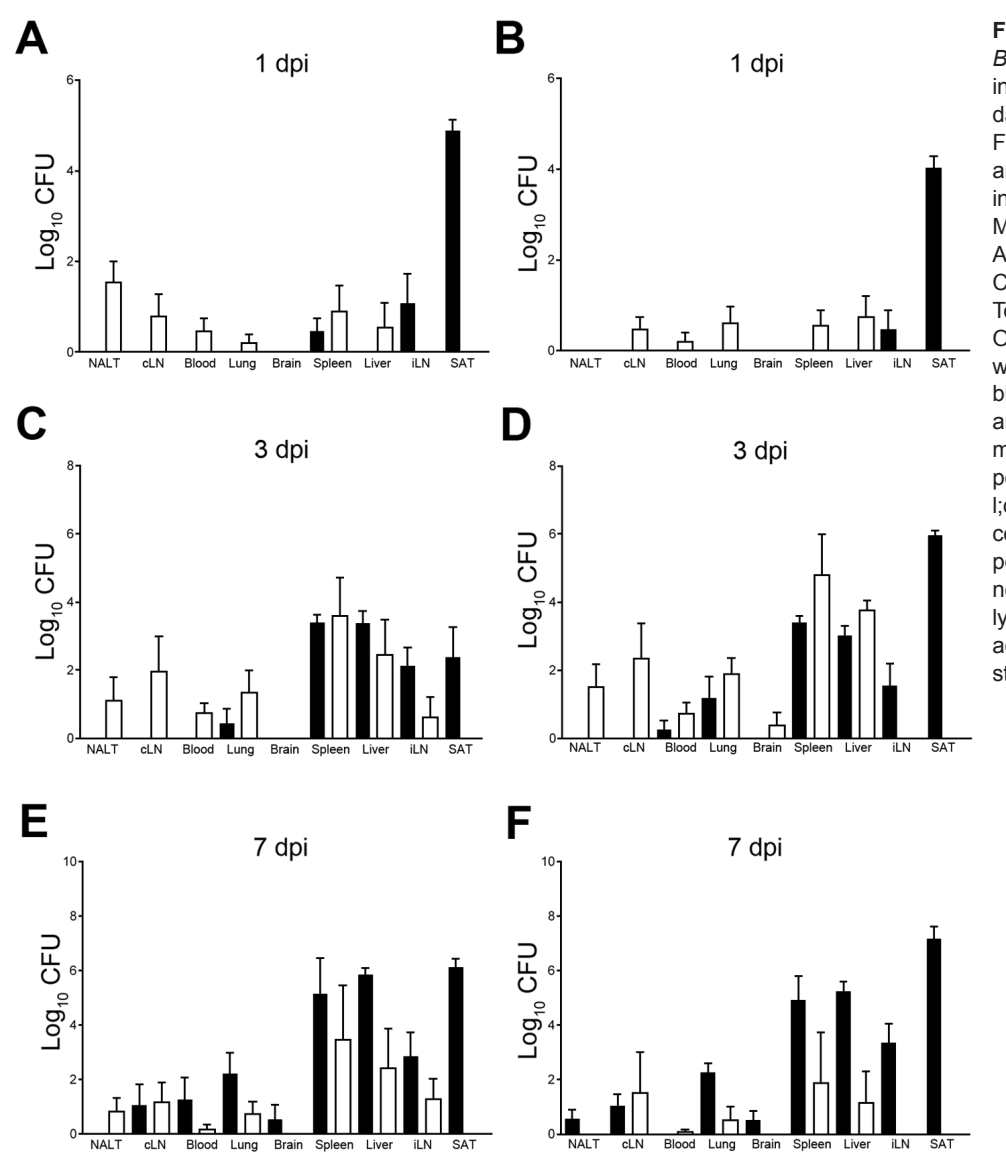
Comparison of *Burkholderia pseudomallei* loads in organs of BALB/c mice at days 1 (A, B), 3 (C, D) and 7 (E, F) after intranasal (white bars) and subcutaneous (black bars) infection with the neurologic isolates MSHR435 (5 × 10^2^ CFU; panels A, C, E) and MSHR1153 (4.5 × 10^2^ CFU; panels B, D, F) , Northern Territory, Australia, October 1989–October 2012. Bacterial loads were assessed in NALT, cLN, iLN, blood, lung, brain, spleen, liver, and SAT at the indicated dpi. Five mice were assessed at each time point. Data are expressed as mean l;og_10_ CFU per tissue ± SEM. cLN, cervical lymph nodes; dpi, days postinfection; iLN, inguinal lymph nodes; NALT, nasal-associated lymphoid tissue; SAT, subcutaneous adipose tissue. Error bars indicate standard error of the mean.

Regardless of the route of infection, dissemination occurred rapidly; bacteria were detected not only at sites of infection but also in draining lymph nodes and spleen by 1 dpi (Figure 2, panels A and B). Bacterial loads continued to increase significantly from 3 dpi (Figure 2, panels C, D) through 7 dpi (Figure 2, panels E, F), with comparable levels in spleen, liver, and lung after intranasal or subcutaneous infection for MSHR435 and MSHR1153. Bacterial loads in the brain of mice infected with MSHR435 and MSHR1153 were low or undetectable within the first week after infection, consistent with the tendency for neurologic signs to develop after 8 dpi. The levels of bacteria recovered from brain after intranasal or subcutaneous infection with MSHR435 or MSHR1153 did not differ between 3 dpi and 7 dpi.

## Discussion

Neurologic melioidosis is a serious, potentially fatal form of *B. pseudomallei* infection (*5*–*7*,*16*). The increased incidence of neurologic melioidosis in persons without recognized risk factors emphasizes the potential public health threat from this form of the disease. The contribution of bacteria- and host-specific factors in the pathogenesis of neurologic melioidosis is poorly understood, as are the potential roles of different modes of infection, such as percutaneous versus respiratory inoculation. Our study evaluated whether *B. pseudomallei* strains isolated from patients with neurologic melioidosis showed higher virulence in animal models of melioidosis than did strains isolated from patients with nonneurologic melioidosis. We found a trend for higher virulence for neurologic isolates than for nonneurologic isolates in an animal model, regardless of route of infection. However, neurotropism was not a unique characteristic of isolates from patients with neurologic melioidosis.

Consistent with the spectrum of clinical presentations of melioidosis, dissemination of *B. pseudomallei* in mice varied substantially. Neurologic signs developed in BALB/c and C57BL/6 mice 8–12 dpi. CNS involvement was not unique to neurologic isolates; we also observed head tilt and limb paralysis in mice infected with nonneurologic isolates. Although neurologic involvement was more commonly associated with intranasal inoculation, signs of CNS infection also occurred after subcutaneous infection. In most instances, subcutaneous infection resulted in localized abscesses in joints of hind limbs and vertebral column, causing hind leg paresis. However, neurologic signs occasionally developed in the absence of lesions at the subcutaneous injection site. When we compared intranasal and subcutaneous infection, we found a similar pattern of dissemination of neurologic isolates, which suggests that no predilection exists for neurologic isolates to invade by the respiratory route.

Our study provides evidence that *B. pseudomallei* isolates from patients with neurologic melioidosis do not demonstrate selective neurotropism in an experimental model. Rather, a subset of *B. pseudomallei* isolates appear to have unique virulence factors that facilitate rapid dissemination to multiple tissues, including the CNS, after both intranasal and subcutaneous exposure. We propose that this group of isolates is associated with severe disease progression and increased rates of death. This finding has valuable public health ramifications for initiating appropriate and timely therapy after exposure to systemically invasive *B. pseudomallei* strains. Studies focused on identifying virulence factors of *B. pseudomallei* that influence systemic spread, together with an improved understanding of the host–pathogen interactions that influence the progression to different forms of melioidosis, will be instrumental in identifying and evaluating future vaccine candidates and novel therapeutics for this potentially life-threatening disease.
